# Reducing stigma and discrimination: Candidate interventions

**DOI:** 10.1186/1752-4458-2-3

**Published:** 2008-04-13

**Authors:** Graham Thornicroft, Elaine Brohan, Aliya Kassam, Elanor Lewis-Holmes

**Affiliations:** 1 Health Service and Population Research Department, Institute of Psychiatry, King's College London, De Crespigny Park, London, UK

## Abstract

This paper proposes that stigma in relation to people with mental illness can be understood as a combination of problems of knowledge (ignorance), attitudes (prejudice) and behaviour (discrimination). From a literature review, a series of candidate interventions are identified which may be effective in reducing stigmatisation and discrimination at the following levels: individuals with mental illness and their family members; the workplace; and local, national and international. The strongest evidence for effective interventions at present is for (i) direct social contact with people with mental illness at the individual level, and (ii) social marketing at the population level.

## Introduction

Widespread discrimination adds to the disability of people with mental illness [1-4]. The basic problem is this: many people with mental illness are subjected to systematic disadvantages in most areas of their lives [5,6]. These forms of social exclusion occur at home, at work, in personal life, in social activities, in healthcare and in the media [7,8].

### From stigma to ignorance, prejudice and discrimination

What is stigma? The concept of stigma is necessary to develop an understanding of experiences of social exclusion, but it is not sufficient to grasp the whole picture, nor to identify what practical steps need to be taken to promote social inclusion. Stigma consists of three related problems:

• The problem of knowledge: *Ignorance*

• The problem of attitudes: *Prejudice*

• The problem of behaviour: *Discrimination*

#### Ignorance

At a time when there is an unprecedented volume of information in the public domain, the level of accurate knowledge about mental illnesses (sometimes called 'mental health literacy') is meagre [9]. A population survey in England, for example, found that most people (55%) believe that the statement 'someone who cannot be held responsible for his or her own actions' describes a person who is mentally ill [10]. Most (63%) thought that fewer than 10% of the population would experience a mental illness at some time in their lives. This ignorance needs to be redressed by conveying more factual knowledge to the general public and also to specific groups such as teenagers, including useful information such as how to recognise the features of mental illness and where to get help [11].

#### Prejudice

Fear, anxiety and avoidance are common feelings both for people who do not have mental illness (when reacting to those who have) and for people with mental illness who anticipate rejection and discrimination and therefore impose upon themselves a form of 'self-stigma' [4]. Although the term 'prejudice' is used to refer to many social groups that experience disadvantage, for example minority ethnic groups, it is employed rarely in relation to people with mental illness. The reactions of a host majority to act with prejudice in rejecting a minority group usually involve not just negative thoughts but also emotions such as anxiety, anger, resentment, hostility, distaste or disgust. Prejudice may more strongly predict discrimination than do stereotypes. A recent study of terms used for mental illness by 14 year old school students in England, for example, found that they used 250 words and phrases, none of which are positive [12].

#### Discrimination

The scientific evidence and the strong message from service users and their advocates indicate that discrimination blights the lives of many people with mental illness, making marriage, childcare, work and a normal social life much more difficult. Actions are needed to specifically redress the social exclusion of people with mental illness and to use the legal measures intended to support all disabled people for physical and mental disabilities on the basis of parity [13]. The evidence from scientific enquiry and consultation with service users is unequivocal: discrimination means that it is harder for people with a mental illness to marry, have children, work or have a social life. This crippling social exclusion needs to be actively addressed. Laws already exist to ensure equality for all people with disabilities.

### Action to support people with mental illness

Empowerment has been described as the opposite of self-stigmatisation [14]. Policy makers can provide specific financial support for ways in which individuals with mental illness can empower themselves or be empowered. Such specific support might include:

• Promoting participation in formulating care plans and crisis plans for people with mental illness.

• Providing cognitive-behavioural therapy for people with mental illness to reverse negative self-stigma.

• Running regular assessments of consumer satisfaction with services.

• Creating user-led and user-run services.

• Developing peer-support worker roles in mainstream mental health care.

• Encouraging employers to give positive credit for experience of mental illness

• Enabling people with mental illness to take part in treatment and service evaluation and research.

### Action to support people with mental illness at work

For some people with mental illness, allowance needs to be made at work for their personal requirements. In parallel with the modifications made for people with physical disabilities, people with mental illness-related disabilities may need what are called 'reasonable adjustments' in relation to the anti-discrimination laws. In practice this can include the following measures:

• Having a quieter work place with fewer distractions for people with concentration problems, rather than, for example, a noisy open plan office, as well as a rest area for breaks.

• Giving more or more frequent supervision than usual to give feedback and guidance on job performance.

• Allowing a person to use headphones to block out distracting noise.

• Creating flexibility in work hours so that they can attend their healthcare appointments or work when not impaired by medication.

• Funding an external job coach for counselling and support and to mediate between employee and employer.

• Providing a buddy/mentor scheme to provide on-site orientation and assistance.

• Writing clear personal specifications, job descriptions and task assignments to assist people who find ambiguity or uncertainty hard to cope with.

• Making contract modifications to specifically allow whatever sickness leave is required by people likely to become unwell for prolonged periods.

• Providing a more gradual induction phase, for example with more time to complete tasks, for those who return to work after a prolonged absence or who may have some cognitive impairment.

• Improving disability awareness in the workplace to reduce stigma and to underpin all other accommodations.

• Reallocating marginal job functions that are disturbing to an individual.

• Allowing use of accrued paid and unpaid leave for periods of illness.

Further, community bodies need to act to promote the social inclusion of people with mental illness. The following initiatives would address discrimination in the workplace and misinformation about mental health issues:

• Employers' federations need to inform employers of their legal obligations under existing disability laws regarding people with mental illness.

• Employers in the health and social care sector, when recruiting, need to make explicit that a history of mental illness is a valuable attribute for many roles.

• Mental health services need to work with employers and business confederations to ensure that reasonable accommodations and adjustments in the workplace are made for people with mental illness.

• The education, health and police authorities need to provide well evaluated interventions to increase integration with, and understanding of, people with mental illness to targeted groups such as schoolchildren, police and healthcare staff.

• Professional training and accreditation organisations need to ensure that mental health practitioners are fully aware of the actual recovery rates for mental illnesses.

### Actions needed at the local level

In local communities or health and social care economies initiatives are needed to promote social inclusion of people with mental illness. These are outlined in Table 1.

**Table 1:** Actions at local level

**Action**	**By**
• Introduction supported work schemes	• Mental health services with specialist independent sector provider
• Psychological treatments to improve cognition, self-esteem and confidence	• Mental health and general health service
• Health and social care explicitly give credit to applicants with a history of mental illness when hiring staff	• Health and social care agencies
• Provision of reasonable adjustments/accommodations at work	• Mental health providers engaging with employers and business confederation
• Inform employers of their legal obligations under disability laws	• Employers' confederation
• Deliver and evaluate the widespread implementation of targeted interventions with targeted groups, including school children, police and healthcare staff	• Education, police and health commissioning and provider authorities
• Provide accurate data on mental illness recovery rates to mental health practitioners	• Professional training and accreditation organisations
• Implementation of measures to support care plans negotiated between staff and consumers	• Mental health provider organisations and consumer groups

### Actions needed at the national level

In national policy a series of changes is necessary that spans government ministries, the non-government and independent sector and service user and professional groups. This is a vision of a long-term attack upon individual and systemic/structural discrimination [6] through a co-ordinated, multi-sectoral programme of action to promote the social inclusion of people with mental illness. Further social marketing approaches, the adaptation of advertising methods for a social good rather than for the consumptions of a commodity, are increasingly often being used [15-17].

In terms of change needed in mental health systems, several elements are necessary. An example is the development of psychological services designed to support people in or seeking work. Many people with mental illness experience demoralisation, reduced self-esteem, loss of confidence, and sometimes depression [18-22]. It is therefore likely that support programmes assisting people with mental illness to gain employment will need to assess whether structured psychological treatment is also needed [23-25]. Second, mental health staff may increasingly see the need to widen their remit from direct treatment provision to also intervening for local populations. Mental health awareness campaigns toward local programmes can be targeted to specific groups [26-28]. In the anti-stigma network of the World Psychiatric Association (called 'Open the Doors'), for example, such interventions have most often been applied to medical staff, journalists, school children, police, employers and church leaders [29-33].

Another key target group is healthcare professionals. Consumers surprisingly often relate that their experiences of general healthcare and mental healthcare staff reveal levels of ignorance, prejudice and discrimination that they find deeply distressing. This has been confirmed by studies in Australia, Brazil, Canada, Croatia, England, Malaysia, Spain and Turkey [34-42]. Based on the principle 'catch them young', several programmes have given anti-stigma interventions to medical students [39,43-46]. As is usual in the field of stigma and discrimination, there is more research describing stigma than assessing which interventions are effective. In Japan, one study found that the traditional medical curriculum led to mixed results: students became more accepting of mentally ill people and mental health services, and more optimistic about the outlook with treatment, but there was no impact on their views about how far people with mental illness should have their human rights fully observed [47]. Positive changes in all of these domains were achieved with a one-hour supplementary educational programme [48].

Interestingly, it seems that psychiatrists may not be in the best position to lead such educational programmes. Studies in Switzerland found no overall differences between the general public and psychiatrists in terms of social distance to mentally ill people [49]. Psychiatry itself tries to walk the narrow tightrope between the physical/pharmacological and psychological/social poles [50]. Clinicians who keep contact with people who are unwell, and who selectively stop seeing people who have recovered, may therefore develop a pessimistic view of the outlook for people with mental illnesses [51]. On balance, there is mixed evidence about whether psychiatrists can be seen as stigmatisers or destigmatisers [52]. Mental health nurses have also been found to have both more and less favourable views about people with mental illness than the general public [36]. Interestingly, nurses, like the general population, tend to be more favourable if they have a friend who is mentally ill, i.e. if there is a perceived similarity and equality with the person affected [53].

What, then, should mental health staff do? Direct involvement in the media is a vital route that professionals can use more often, with proper preparation and training. They also need to set their own house in order by promoting information within their training curricula, continuing professional development (continuing medical education) and relicensing/revalidation procedures which ensures that they have accurate information, for example, on recovery [54].

Further, practitioners need in future to pay greater attention to what consumers and family members say about their experiences of discrimination, for example in relation to work or housing. Staff can also work directly with consumers to combat social exclusion, for example by opposing repressive or regressive mental health laws [55]. Third, it is clear that consumer groups increasingly seek to change to the terms of engagement between mental health professionals and consumers, and to move from paternalism to negotiation [14]. Vehicles to support shared decision-making include: crisis plans [56] which seem able to reduce the frequency of compulsory treatment [57], advance directives [58], shared care agreements [59] and consumer-held records [60]. The key fact is that many consumers want direct participation in their own care plans [61].

Going into the public advocacy domain, staff in mental health systems may well develop in future a direct campaigning role. A practical approach is for local and national agencies to set aside their differences and to find common cause. In various areas, such co-ordinating groups are called forums, peak bodies, alliances or consortia. What they have in common is a recognition that what they can achieve together, in political terms, is greater that their individual impact. Core issues able to unite such coalitions are likely to include parity in funding, the use of disability discrimination laws for people with mental illness-related disabilities, and the recognition of international human rights conventions in practice [62-65]. The actions needed at a national level are summarised in Table 2.

**Table 2:** Actions at national level

**Action**	**By**
• Use a social model of disability that refers to human rights, social inclusion and citizenship	• Governments and non governmental organisations (NGOs) to change core concepts
• Apply the anti-discrimination laws to give parity to people with physical and mental disabilities	• Parliament and government
• Inform all employers of their legal obligations under these laws	• Ministry of Employment or equivalent
• Interpret anti-discrimination laws in relation to mental illness	• Judiciary and legal profession
• Establish service user speakers' bureaux to offer content to news stories and features on mental illness	• NGOs and other national level service user groups
• Provide and evaluate media watch response units to press for balanced coverage	• Statutory funding for NGOs to provide media watch teams
• Share between countries the experience of disability discrimination acts	• Legislators, lawyers, advocates and consumer groups
• Understand and implement international legal obligations under binding declarations and covenants	• NGOs to communicate legal obligations of all stakeholders and health and social care inspection agencies to audit how far these obligations are respected in practice
• Audit compliance with codes of good practice in providing insurance	• Associations of Insurers with Service User organisations and mental health NGOs
• Providing economic incentives rather than disincentives to disabled people ready to return to work	• Employment Ministries to introduce new and flexible arrangements for disabled people to work with no risk to their income
• Change laws to allow people with a history of mental illness to serve on juries with a presumption of competence	• Justice ministries to amend the laws relating to jury service

### Action at the international level

What action is necessary at the international level? Such contributions, so far removed from the everyday lives of people, may be hardly noticeable unless they are very sharply focussed and coherent. Setting international standards for national polices could be one useful intervention. For example the World Health Organisation (WHO) has published standards to guide countries in producing and revising mental health laws [66]. The standards cover advice on:

• access to care

• confidentiality

• assessments of competence and capacity

• involuntary treatment

• consent

• physical treatments

• seclusion

• restraint

• privacy of communications

• appeals against detention

• review procedures for compulsory detention [66]

At present, 25% of countries worldwide do not have mental health legislation and half of those that do enacted their laws over 15 years ago. Generally, lower income countries are more likely to have older legislation [67].

In the European Union, for example, anti discrimination laws are now mandatory under the Article 13 Directive [68]. Such laws must make illegal all discrimination in the workplace on grounds that include disability, and also set up institutions to enforce these laws. The time is therefore right is share experiences between different countries on how successful such laws have been to reduce discrimination against people with mental illness and to understand more clearly what is required both for new legislation elsewhere and for amendments to existing laws that fall short of their original intentions.

International organisations such as the WHO can also contribute towards better care and less discrimination by indicating the need for national mental health policies and by giving guidance on their content. In 2005, for example, only 62% of countries in the world had a mental health policy [67]. In Europe, health ministers have signed a Mental Health Declaration and Action Plan which sets the following priorities:

• foster awareness of mental illness

• tackle stigma, discrimination and inequality

• provide comprehensive, integrated care systems

• support a competent, effective workforce

• recognise the experience and knowledge of services users and carers [65,69,70].

## Conclusion

The strongest evidence at present for active ingredients to reduce stigma pertains to direct social contact with people with mental illness, which has been shown to be effective in relation to police officers, school students, journalists and the clergy [33,71-73]. At the national level, there is emerging evidence that a carefully co-ordinated approach based on using social marketing techniques, namely advertising and promotional methods designed to achieve a social good rather than sales of a commodity, have produced benefits in Australia, New Zealand and Scotland [16,17,74]. The challenge in the coming years will be to identify which interventions (whether directed towards knowledge, attitudes or behaviour) are most cost-effective in reducing the social exclusion of people with mental illness.
